# Adolescent body satisfaction: the role of perceived parental encouragement for physical activity

**DOI:** 10.1186/1479-5868-6-90

**Published:** 2009-12-09

**Authors:** Jennifer S Savage, Jennifer M DiNallo, Danielle Symons Downs

**Affiliations:** 1 Department of Nutritional Sciences and Center for Childhood Obesity Research, The Pennsylvania State University, University Park, PA 16802, USA; 2 Departments of Kinesiology, 266 Recreation Building, The Pennsylvania State University, University Park, PA 16802, USA

## Abstract

**Background:**

Parents play an important role in the development of children's health behaviors, but less is known about the role of parental encouragement for physical activity (PA) on youth PA behavior and body image satisfaction. The purposes of this study were to: (1) longitudinally assess whether adolescent PA at age 15 mediates the effect of perceived parental encouragement for PA at age 15 for predicting adolescent body satisfaction at age 16, while controlling for body mass index (BMI), and (2) examine the extent to which adolescent sex moderates this association.

**Methods:**

Participants were 379 boys and girls assessed at 15 and 16 years of age, who completed surveys as part of a larger longitudinal study in their health/physical education classes in a school district in Central Pennsylvania. Participants completed measures of their perception of parental encouragement for PA, PA behavior, body satisfaction, and height and weight to calculate BMI at age 15 and 16 (i.e., 10^th ^and 11^th ^grades). Pearson correlations were used to examine the association among the study variables and hierarchical regression analyses were used to predict body satisfaction at age 16.

**Results:**

Perceived encouragement for PA from fathers, but not mothers, at age 15, was significantly associated with adolescent PA at age 15 and body satisfaction scores at age 16. Adolescents reporting higher PA behavior and perceived encouragement for PA from fathers at age 15 had higher body satisfaction scores at age 16. Moreover, adolescent PA at age 15 mediated the association between perceived fathers' encouragement for PA at age 15 and adolescent body satisfaction at age 16, when controlling for BMI. Examining the moderating effect of adolescent sex on this association revealed that adolescent PA no longer mediated the association between perceived encouragement for PA from fathers and adolescent body satisfaction, and sex moderated this association.

**Discussion:**

These findings suggest that, regardless of adolescent BMI, fathers may play an instrumental role in adolescents' body image satisfaction by positively influencing PA behavior. However, the influence of perceived encouragement for PA from fathers on adolescent body satisfaction and PA behavior may differ for boys and girls.

## Background

With increasing rates of obesity [1] and physical inactivity [2-4] among adolescents, it is not surprising that many adolescents, especially young girls, are dissatisfied with their bodies [5]. One potential protective factor against body dissatisfaction is increased physical activity (PA). Specifically, PA has been positively associated with body image satisfaction, self-esteem, and overall physical self-worth, independent of changes in body mass index (BMI) [6-8]. In other words, even in the absence of changes in body mass, adolescents may benefit psychologically from increases in PA. While it is evidenced that physically active adolescents become physically active adults [9,10], children tend to become less active as they move through adolescence and approach puberty [11-13]. Therefore, it is important to identify existing modifiable factors that may promote the maintenance of PA during adolescence and ultimately encourage body satisfaction, which may also help to inform the development of youth PA interventions. Importantly, improving body satisfaction may also reduce the risk of disordered eating in adolescents who are at high risk for these maladaptive eating behaviors [14].

Parents play an important role in the development of youth PA behavior [15]. Specifically, several forms of parental support have been positively associated with children's PA including direct support (e.g., facilitating activity via transportation, enrolling children in sports) [16,17], serving as models (e.g., parents being active themselves) [18-20], and motivational or emotional support (e.g., encouragement) [21-23]. To date, much of this literature has focused on the influence of parental modeling despite findings that perceived parental PA encouragement may be a stronger predictor of child PA behavior [24,25]. Moreover, little is known about the differential effect of mothers' versus fathers' encouragement for PA on adolescent boys' and girls' PA behavior over time. However, there is some evidence that parent PA and encouragement may influence activity patterns of girls to a greater extent than boys [26-28]. Lastly, while it has been suggested that body dissatisfaction emerges from pressure from parents, peers, or the media to maintain a thin or ideal body size [29], the effect of direct parental (maternal or paternal) encouragement for PA on body image satisfaction has not been explored.

Therefore, the purposes of this study were to: (1) longitudinally assess whether adolescent PA at age 15 mediates the effect of parental encouragement for PA at age 15 for predicting adolescent body satisfaction at age 16, while controlling for body mass index (BMI), and (2) examine the extent that adolescent sex moderated the mediation of adolescent PA on the association between parental encouragement for PA and body satisfaction. We hypothesized that perceived fathers' and mothers' encouragement for PA would positively predict adolescent PA and body satisfaction, and that greater adolescent PA would explain this relationship between parental encouragement for PA at age 15 and adolescent body satisfaction at age 16 [6,8,21]. We also expected that parental encouragement would influence PA behavior of girls to a greater extent than boys, especially among same sex parent and child [26].

## Methods

### Participants

Participants were 379 adolescent boys (*N *= 221; *M *age = 15.0 years, *SD *= 0.6; *M *BMI = 22.7 kg·m^-2^, *SD *= 3.6) and girls (*N *= 166; *M *age = 15.0 years, *SD *= 0.8; *M *BMI = 22.2 kg·m^-2^, *SD *= 3.7) who were part of a larger longitudinal study designed to assess the determinants of healthy lifestyle habits among middle and high school students in a school district in Central Pennsylvania. Most of the participants were Caucasian (83%), followed by Asian American (4%), African American (3%), Hispanic American (2%), American Indian (1%), and other (3%). The University's Institutional Review Board and the School District's Board of Education approved this study. Written informed consents were completed by both students and parents. Data was collected during the Spring semester for a two-year period, annually. At each time of assessment, students completed a series of self-report questionnaires during their health and Physical Education classes, lasting approximately 45 minutes.

### Measures

The *Personal History Questionnaire *assessed the participants' sex, age, grade in school, race/ethnicity, and self-reported height and weight. BMI was calculated by converting self-reported weight from pounds to kilograms, and by transforming self-reported height from inches to meters (kg·m^-2^). Self-reported BMI has been found to be highly correlated with actual BMI in adolescents [30].

#### Perceived Parental Encouragement for Physical Activity

Due to the paucity of research in this area and the limited assessment instruments; two author-developed questions were used to assess perceived parental encouragement to be physically active. The questions, (1) "*How much does your mother/guardian encourage you to be physically active*" and (2) "*How much does your father/guardian encourage you to be physically active*", were on a 7-point Likert scale ranging from 0 (no encouragement) to 7 (a lot of encouragement). Values for maternal and paternal encouragement were significantly correlated (*r *= 0.62, *p *< 0.001). In pilot work by the authors, the 2-item measure of parental encouragement for PA was validated against a measure of support (i.e., two items assessing Subjective Norm from the Theory of Planned Behavior) [31]. Specifically, perceived encouragement for PA from mothers' (*r *= .56, *p *< .01) and fathers' (*r *= .63, *p *< .01) was significantly associated with Subjective Norm items assessing mothers' and fathers' encouragement to be moderately active at least 60 minutes on most days of the week.

#### Physical Activity

The Leisure-Time Exercise Questionnaire [LTEQ] developed by Godin & Shephard [32] was used to assess the frequency of exercise behavior. Three questions were asked: "How often in a typical week have you engaged in (1) *mild *(e.g. easy walking), (2) *moderate *(e.g. brisk walking), and (3) *strenuous *(e.g. running, aerobic dance) leisure-time exercise, performed for at least 15 minutes?" A weekly PA index in metabolic equivalents (METs) was generated by multiplying the estimated rate of energy expenditure by the reported frequency of participation: (3 * # *mild *bouts) + (5 * # *moderate *bouts) + (9 * # *strenuous *bouts). The LTEQ is a valid and reliable index of PA behavior in youth [33].

#### Body Satisfaction

The Body Areas Satisfaction Scale [BASS] is a 9-item subscale of the Multidimensional Body Self-Relations Questionnaire developed by Cash [34]. Participants rate the degree of satisfaction with specified body parts (e.g., face, thighs) and overall muscle tone, weight, height, and appearance using a 5-point Likert scale ranging from 1 (very dissatisfied) to 5 (very satisfied). Therefore, a higher score on the BASS represents greater satisfaction with one's body. The 1-month test-retest reliability ranges from 0.74 - 0.86 [35]. The internal consistency of the total BASS score in this study was good (α = .86).

### Data Analysis

Pearson correlations were used to examine the associations among the study variables for the total sample and by the sex of the adolescents. All statistical tests were conducted with alpha set at .05. Two independent regression analyses were conducted to examine separately the contribution of perceived mother's and father's encouragement for PA at age 15 for predicting adolescent body image satisfaction at age 16. Next, a 4-step hierarchical regression analyses was used to test mediation [36] adjusting for adolescent BMI at study entry (age 15) to examine the contributions of adolescent PA as a mediator of the association between perceived parental encouragement for PA and adolescent body image satisfaction for the total sample. To establish mediation, four equations were tested: 1) parent encouragement for PA predicting adolescent body satisfaction; 2) parent encouragement for PA predicting the mediator (adolescent PA); 3) adolescent PA predicting body satisfaction; and 4) adolescent PA and parent encouragement of PA predicting adolescent body satisfaction. To confirm mediation, Models 1-3 need to be significant, but when adolescent PA is added to model 4, encouragement for PA is no longer a significant predictor of body satisfaction. Lastly, a test for moderated mediation was used to examine the moderating effect of sex on this association [37].

## Results

Descriptive statistics reveal that mean BMI at age 15 (boys *M *= 22.7 kg·m^-2^, *SD *= 3.5; girls *M *= 22.2 kg·m^-2^, *SD *= 3.7) was similar to the National Health and Nutrition Examination Survey III (NHANES III) data in which 15-year-old boys' and girls' mean BMI was 22.3 kg·m^-2 ^and 23.2 kg·m^-2^, respectively [38]. Also similar to National data, 28.4% of all participants were overweight (BMI ≥ 85^th ^percentile) at 15 years, with 34.2% of boys and 21.3% of girls falling above the 85^th ^percentile [39]. As previously reported [11,13], mean self-reported PA (weekly METs) significantly decreased from age 15 to age 16 for both boys (*t *(172) = 6.92, *p *< .001; age 15 *M *= 143.3, *SD *= 71.1; age 16 *M *= 114.7, *SD *= 62.6) and girls (*t *(120) = 2.50, *p *< .01; age 15 *M *= 104.6, *SD *= 63.7; age 16 *M *= 89.5, *SD *= 50.9), with boys self-reporting significantly more PA at age 15 (*F *(1,350) = 27.97, *p *< .001) and age 16 (*F *(1, 315) = 14.35, *p *< .001) compared to girls. Lastly, adolescents perceived that both their mothers (*M *= 4.9, *SD *= 1.8, range 0 - 7) and fathers (*M *= 4.9, *SD *= 2.1, range 0 - 7) encouraged them to be physically active, with boys reporting significantly higher perceived encouragement for PA from their fathers, *F *(1, 376) = 6.07, p < .01, compared to girls. No difference in perceived parental encouragement for PA from mothers was observed between boys and girls (*p *> .05).

### Correlations for Study Variables for Total Sample and By Sex

As predicted, perceived fathers' (*r *= 0.17, *p *< 0.001) and mothers' (*r *= 0.17, *p *< 0.001) encouragement for PA at age 15 was positively associated with adolescent PA at age 15. However, examining this association by adolescent sex revealed that mothers' (*r *= 0.22, *p *< .01) and fathers' (*r *= 0.16, *p *< .05) encouragement was associated with daughters' PA, but not sons' PA. Moreover, self-reported adolescent PA at age 15 was positively associated with adolescent body satisfaction at age 16 (*r *= 0.14, *p *< .01). In partial support of our first hypothesis, Pearson correlations among the predictors (perceived encouragement for PA and adolescent PA at age 15) and outcome (body satisfaction at age 16) revealed that perceived encouragement for PA from fathers (*r *= 0.11, *p *< 0.05), but not mothers (*r *= 0.07, *p *> .05), was positively associated with adolescent body satisfaction one year later.

Correlations were also examined by sex revealing that fathers' encouragement for PA at age 15 was only positively associated with body satisfaction reported by their sons (*r *= 0.18, *p *< 0.01), not daughters (*r *= -0.06, *p *> .05) one year later. Adolescent BMI at age 15 was not associated with fathers' (*r *= .01, *p *> .05) or mothers' (*r *= -.01, *p *> .05) encouragement for PA at age 15, or self-reported adolescent PA for the total sample (*r *= .03, *p *> .05) at age 15. Lastly, adolescent body satisfaction was inversely associated with adolescent BMI (*r *= - 0.13, *p *< 0.05). Importantly, this significant inverse association was observed independently for boys (*r *= - 0.19, *p *< 0.01), but not girls (*r *= -.12, *p *> .05). In other words, adolescent boys with higher BMIs at age 15 reported lower body satisfaction one year later, but this same association was not observed for girls.

### Mothers' Encouragement for PA Predicting Body Satisfaction among Adolescents

In contrast to our hypothesis, perceived mothers' encouragement for PA at age 15 was not a significant predictor of adolescent body image satisfaction at age 16; therefore, mothers' encouragement was not considered in further analyses.

### Fathers' Encouragement for PA Predicting Body Satisfaction among Adolescents and by Sex

A hierarchical regression analysis predicting adolescent body satisfaction at age 16 with fathers' encouragement for PA and adolescent PA at age 15, adjusting for adolescent BMI at study entry (age 15) is shown in Table 1. BMI was included as a covariate due to its significant association with adolescent body satisfaction. The 4-step hierarchical regression analysis testing mediation, controlling for BMI, revealed that adolescent PA (*β *= .14, *p *= .01) mediated the association between perceived fathers' encouragement for PA at age 15 and adolescent body satisfaction at age 16 (*z *= 2.02, *p *< .05). In step 1, perceived fathers' encouragement for PA at age 15 significantly predicted adolescent body satisfaction one year later (*β *= .11, *p *< .05). In Step 2, perceived fathers' encouragement for PA at age 15 significantly predicted concurrent adolescent PA (*β *= .17, *p *< .01). In Step 3, adolescent PA at age 15 significantly predicted adolescent body satisfaction at age 16 (*β *= .14, *p *< .01). Finally, according to Baron and Kenney [36], full mediation was confirmed when a regression analysis was performed with fathers' encouragement for PA and adolescent PA behavior significantly predicting adolescent body satisfaction one year later (*β *= .13, *p *< .05). Importantly, when adolescent PA behavior was added to the regression, fathers' encouragement for PA was no longer significant (*p *> .05).

**Table 1:** Mediation Model using Hierarchical Regression Analyses to Predict Adolescents' Body Satisfaction at age 16 with Fathers' Encouragement for Physical Activity and Adolescents' Leisure-time Physical Activity, Adjusting for Adolescents' Body Mass Index at age 15

Variable	*F *change	*Df*	*R*^2 ^change	*β*^1^	*β*^2^
*Step 1: Predicting Age 16 Adolescents' Body Satisfaction with Fathers' Encouragement for Physical Activity Measured at Age 15*					

Block 1	6.54**	326	0.02		
BMI				-0.14**	-0.14**
Block 2	3.82*	325	0.01		
Father encouragement PA					0.11*

*Step 2: Predicting Age 15 Adolescents' Leisure-time Exercise Behavior with Fathers' Encouragement for Physical Activity Measured at Age 15*					

Block 1	0.36	309	0.01		
BMI				0.03	0.03
Block 2	8.65**	308	0.03		
Father encouragement PA					0.17**

*Step 3: Predicting Age 16 Adolescent Body Satisfaction with Adolescent Leisure-time Exercise Behavior Measured at Age 15*					

Block 1	5.61*	308	0.02		
BMI				-0.13*	-0.14**
Block 2	6.15**	307	0.02		
LTEQ					0.14**

*Step 4: Predicting Age 16 Adolescent Body Satisfaction with Fathers' Encouragement for Physical Activity and Adolescent Leisure-time Exercise Behavior Measured at Age 15*					

Block 1	6.59**	300	0.02		
BMI				-0.15**	-0.15**
Block 2	4.18*	298	0.03		
Father encouragement PA					0.08
LTEQ					0.13*

*Note*.β^1-2 ^= standardized regression coefficients; *Df *= degrees of freedom. *p < .05; **p < .01; Abbreviations. BMI = body mass index; LTEQ = Leisure-Time Exercise Questionnaire

**Figure 1:**
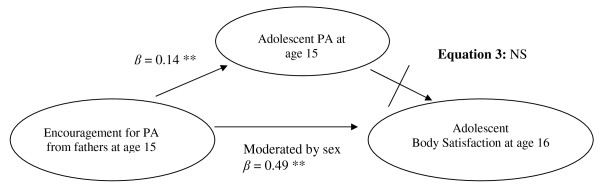
**Moderated mediation model for the associations between fathers' encouragement for PA at age 15 and adolescent body satisfaction at age 16 as mediated by adolescent PA at age 15 and moderated by adolescent sex**. After adjusting for adolescent sex and BMI: 1) encouragement for PA from fathers predicted adolescent PA (adolescent sex did not moderate this association); 2) fathers encouragement for PA predicted adolescent PA as moderated by adolescent sex; however, 3) adolescent PA was no longer a significant predictor of body satisfaction. *p < .05; **p < .01.

**Figure 2:**
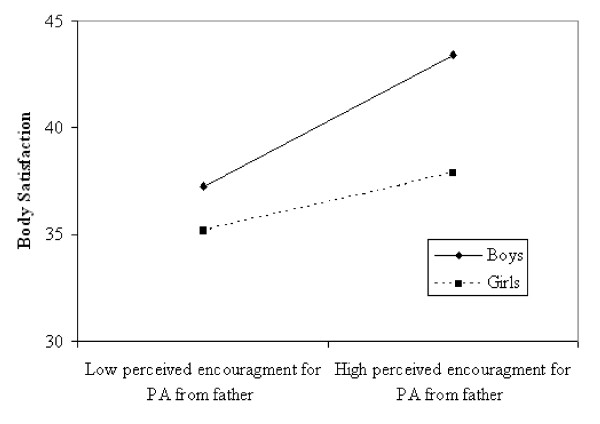
**Illustration of the effect of fathers' encouragement for PA at age 15 on adolescent body satisfaction at age 16 as moderated by adolescent sex (2-way interaction), adjusting for adolescent BMI**. Solid line represents boys; dashed line represents girls.

Hierarchical regression analyses were also used to examine the extent to which adolescent sex moderated the mediation of PA on the association between parental encouragement for PA and body satisfaction, based on the finding that associations varied by adolescent sex. As depicted in Figure 1, perceived encouragement from fathers predicted adolescent body satisfaction one year later, and this association was moderated by sex (*β *= .49, *p *< .01). Specifically, perceived encouragement from fathers only predicted body satisfaction for boys (see Figure 2). In addition, perceived encouragement for PA from fathers predicted adolescent PA at age 15 (*β *= .14, *p *< .01); however, this association was not moderated by sex (*β *= .04, *p *> .05). Lastly, adolescent PA was no longer a significant predictor of body satisfaction one year later, after adjusting for sex (*β *= .05, *p *> .05). Thus, adolescent PA did not mediate the association between perceived encouragement for PA and adolescent body satisfaction one year later, after adjusting for sex.

## Discussion

The purpose of the present study was to examine the extent to which perceived parental encouragement for PA at age 15 predicted adolescent body satisfaction at age 16, and whether adolescent PA at age 15 mediated this association. Findings from the present study support the general premise that parents have a strong influence on the PA habits of their children [15,21-23]. However, this is the first study to show that perceived encouragement from fathers', but not mothers, predicted adolescent body satisfaction. Specifically, fathers play an important role in promoting body satisfaction by encouraging adolescents to be physically active. Importantly, findings from the present study also suggest that parental influence on adolescent PA may vary by sex, such that perceived encouragement for PA from fathers, but not mothers, predicted body satisfaction among their sons, but not daughters.

Consistent with previous findings [26-28], results from the present study reveal that maternal and paternal support for PA influence adolescent boys and girls differently. Specifically, encouragement from fathers, but not mothers, was positively associated with greater body satisfaction among their sons; whereas, both fathers' and mothers' encouragement for PA predicted adolescent PA one year later. One potential explanation is that mothers and fathers may use different strategies to encourage their sons and daughters to be active which may differentially impact adolescent PA and body satisfaction. For example, Davison et. al. [16] found that mothers were more likely to provide logistic support such as supporting their 9-year-old daughters at sporting events, whereas fathers were more likely to use their own behavior to encourage PA behavior. In contrast to our findings, they also reported that only mothers' logistic support for PA, not fathers', predicted higher levels of PA among their daughters. Their study was also limited to a sample of girls and did not examine associations between body satisfaction and parental encouragement for PA behavior. However, Davison and colleagues [16] multidimensional scale of parental encouragement for PA (Parent Activity Support Scale) may be a better method for capturing parental encouragement, but unfortunately it was not available at the time the present study was conceptualized. Thus, we are unable to make actual parallel comparisons to their findings. Collectively, our findings suggest that additional longitudinal research among adolescent boys and girls is needed examining different types of parental encouragement to better understand the influence of parental encouragement on adolescent PA, as well as the link with body satisfaction.

While previous research has found that PA and body satisfaction are positively associated [6,40] and that parental encouragement for PA is associated with child PA [21-23], this is the first study to longitudinally examine the mediating influence of adolescent PA behavior on the association between parental encouragement for PA and adolescent body satisfaction one year later. Moreover, strength of the present study is that mothers' and fathers' encouragement for PA on adolescent PA and subsequent body satisfaction were examined separately. Lastly, findings from the present study indicate adolescent sex may moderate associations among parental encouragement for PA, adolescent PA, and body satisfaction; thus, future work should also consider examining the moderating effects of sex between same sex and opposite sex parents.

Although our findings contribute to the parental support of adolescent PA literature by examining associations with reported PA behavior and body satisfaction, there were study limitations. First, it should be noted that this sample is rather homogenous in that most participants were Caucasian and from middle-to-high income families. To generalize the findings to more diverse populations, further research is needed to replicate these findings. Moreover, our small study sample size may explain the lack of support for our second hypothesis that adolescent sex would moderate the association between adolescent PA and body satisfaction. Second, although PA was assessed with a valid and reliable measure, it was nonetheless obtained through self-report methods, which is inherently biased by social desirability. Further research assessing youth PA with objective measures (i.e., pedometers and accelerometers) may provide a more accurate and descriptive assessment of the associations among the variables. Lastly, parent report of encouragement for adolescent PA was not assessed; thus additional research is warranted.

To date, only one other study has been located examining the association between fathers' and mothers' encouragement for PA and adolescent BMI in boys and girls. Similar to our findings, Springer and colleagues [23] found that perceived family encouragement to be physically active and BMI were not associated in a sample of 6^th ^grade girls. In contrast to the present study, it should be noted that Springer and colleagues assessed perceived family support rather than maternal and parental support separately. Thus, additional research is needed to confirm our study findings.

While the present study found that only boys' body satisfaction was inversely associated with BMI, Stice and Whitenton [29] found that BMI was inversely associated with body satisfaction one year later for both boys and girls. One potential explanation for this inconsistent finding is that BMI was self-reported and girls may be more likely to underreport their weight compared to boys [41].

## Conclusion

Findings from this study highlight that regardless of BMI, fathers' encouragement for PA influences adolescent body satisfaction one year later, but only due to the influence on adolescent PA. In other words, fathers may play an instrumental role in adolescents' body image satisfaction by positively influencing PA behavior. Interestingly, this model is in contrast to previous research supporting the influence of mothers as the primary encouragers for PA [16,42]. It should also be noted that the mediating effect of adolescent PA was no longer significant after examining the moderating effect of sex; however, the relatively small sample size may explain the lack of support for moderated mediation. Future research is needed to test the assumption that fathers' encouragement for PA during adolescence may play a significant role in positively influencing PA behavior and body satisfaction to better understand the roles that both mothers and fathers play in their children's PA behavior and body satisfaction. Moreover, it is well evidenced that parents have greater influence over offspring attitudes and behaviors, including PA and other health behaviors, when they are pre-adolescent, but peer influence becomes more pronounced during adolescent [43]. Therefore, it is plausible that age and development of the child may also moderate the effect of parental encouragement for PA on body image through child PA; this requires further study.

## Competing interests

The authors declare that they have no competing interests.

## Authors' contributions

DSD conceived of the study and contributed to the study design. All authors contributed to the conceptual approach, statistical analyses, interpretation of the results and manuscript preparation. All authors participated in data collection. All authors read and approved the final manuscript.
